# The role of packaged water in meeting global targets on improved water access

**DOI:** 10.2166/washdev.2017.155

**Published:** 2017-04-07

**Authors:** Sridhar Vedachalam, Luke H. MacDonald, Elizabeth Omoluabi, Funmilola OlaOlorun, Easmon Otupiri, Kellogg J. Schwab

**Affiliations:** 1 Johns Hopkins Water Institute, Johns Hopkins Bloomberg School of Public Health, 615 N Wolfe St, E6638, Baltimore, MD 21205, USA; 2 **Elizabeth Omoluabi** Center for Research, Evaluation Resources and Development, Ife, Nigeria And University of Western Cape, Cape Town, South Africa; 3 **Funmilola OlaOlorun** Department of Community Medicine, College of Medicine, University of Ibadan, Ibadan, Nigeria; 4 **Easmon Otupiri** School of Public Health, Kwame Nkrumah University of Science and Technology, Kumasi, Ghana

**Keywords:** bottled, Ghana, improved, Nigeria, packaged water, sachet

## Abstract

Packaged water (as either refill, bottled, or sachet water) has become an important element of water security in many low- and middle-income countries, owing to poor reliability and lack of piped water infrastructure. However, over time and across countries, the Demographic and Health Surveys monitoring program has inconsistently classified packaged water components as either improved or unimproved. Using data collected as part of the Performance Monitoring and Accountability 2020 (PMA2020) surveys on water options in nine study geographies across eight countries, we identified five geographies where packaged water constituted one of several options for 5% or more of users. In this study, four scenarios were designed in which packaged water components were variously classified as either improved or unimproved. Unimproved water use was highest in scenarios where sachet or refill water was classified as an unimproved source. Across the four scenarios, the difference in the use of unimproved water as the main option was highest (65%) in Nigeria (Lagos). That difference increased to 78% when considering all regular options. The development of these scenarios highlights the importance of classifying a source as improved or unimproved in the overall metric that indicates progress at national and international levels.

This is an Open Access article distributed under the terms of the Creative Commons Attribution Licence (CC BY 4.0), which permits copying, adaptation and redistribution, provided the original work is properly cited (http://creativecommons.org/licenses/by/4.0/

## INTRODUCTION

**Figure 1:**
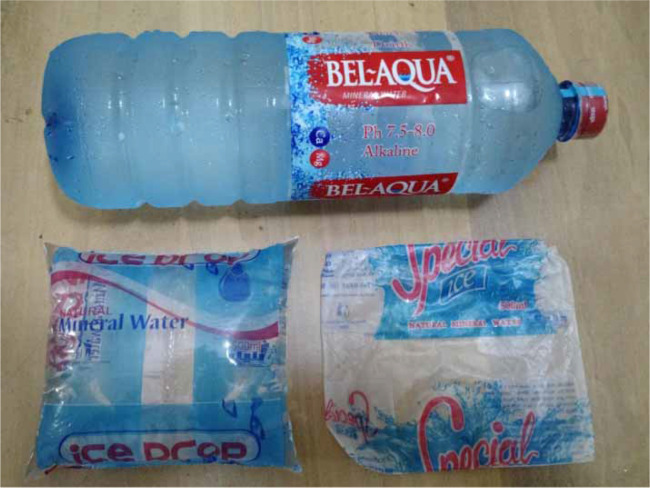
Bottled water in a 1.5 L packaging and two 500 mL sachet water packs sold under various brands in GH (photo credit: author).

Lack of access to direct piped water supply in many low- and middle-income countries (LMICs) has led residents to seek alternative sources (Bakker *et al*. 2008). Among sources that are actively promoted by governments, businesses, and local entrepreneurs to meet this shortcoming is water packaged in disposable plastic bottles and small sachets, as well as in large refillable containers. In the rapidly growing cities of LMICs, packaged water (an umbrella term that includes bottled, sachet, and refill water) bridges the needs unmet by public infrastructure, and has seen an enormous increase in its usage (Kassenga 2007; Stoler 2012). In rapidly growing cities of the south such as Chennai, India (metropolitan population: 10 million), a shortfall of about 200 million liters of piped water supply is met by 5 million units of sachet water, 75,000 units of 1 liter bottled water, and more than 100,000 units of refill water daily (Venkatachalam 2015). No place is as intimately tied to the birth and proliferation of the sachet water industry as West Africa, most notably Nigeria (NG) and Ghana (GH). In a majority of GH's ten regions, reliance on sachet water (i.e., small ~500 mL bagged water sold individually or in packs) as the primary drinking source increased by about 5% between 1998 and 2008 (Stoler et al. 2012b). The largest increase, however, occurred in the Greater Accra region where sachet water use rose from almost zero to 35% over the ten-year period. A competitive market and easy availability of packaged water has made it a popular choice, especially among the highly mobile urban population (Figure 1).

Even as packaged water use has grown, its safety has remained in question however. Samples collected in high-income countries have shown elevated bacterial levels compared to tap water samples (Raj 2005). There is an extensive literature on the microbiological quality of packaged water at various points in the distribution channel, but is heavily focused on point-of-use quality. Packaged water sold in GH and NG is of varying quality, depending on the type of packaging. While bottled water in these two countries was generally found to show no or lower rates of contamination (Obiri-Danso *et al* 2003; Oyelude & Ahenkorah 2012; Igbene-ghu & Lamikanra 2014), factory-bagged sachet water samples showed pathogenic contamination in as little as 5% to as much as 60 to 70% of the samples (Obiri-Danso et al. 2003; Mgbakor *et al* 2011; Oyelude & Ahenkorah 2012). When tested for a range of parasitic protozoa such as *Microsporidia* sp. and *Cryptosporidium parvum*, 77% of the sachet samples were found to be contaminated (Kwakye-Nuako *et al* 2007).

The worst performers were the hand-filled hand-tied polythene bags. Fecal or total coliforms were found in nearly half (Obiri-Danso *et al*. 2003) to all of the samples tested (Okioga 2007; Oyelude & Ahenkorah 2012).

Although most studies have evaluated the quality of bottled or sachet water that is *inside* the packaging, there is evidence to suggest that an equally important component of water quality might be what is *outside* the packaging, especially during distribution and point-of-use. Egwari *et al*. (2005) identified enteric pathogens and *Escherichia coli* in samples collected from cooling receptacles used in the sale of sachet water, the surface of sachets, and from melted water used to cool those sachets. In light of these results, it is no comfort to know that sachet water is more commonly sold as 'pure water' in GH and NG (Akunyili 2003).

Of course, not all studies point to sub-standard sachet water quality. Ahimah & Ofosu (2012) report complete compliance with national standards when evaluating sachet water found in the streets of a large city in southeastern GH. Sachet water use is also associated with a lower likelihood of diarrhea in children and higher levels of self-reported health in women (Stoler *et al*. 2012a). A recent study reported that packaged water (sachet or bottled) provides protection against point-of-consumption E. coli contamination as compared to piped water, providing public health benefits (Wright *et al*. 2016). The ambiguous safety of packaged water perhaps played a role in how it has been classified according to the Demographic and Health Surveys (DHS), a USAID-led initiative that has set global benchmarks for drinking water and sanitation standards. DHS results, in turn, feed into the WHO/UNICEF's Joint Monitoring Programme (JMP) that tracks progress on water and sanitation access.

DHS classifies water sources as improved or unimproved based on a ladder that hierarchically places each water source based on the likelihood of the source being contaminated. In addition to the main source, respondents are asked to list a backup source as well. Placing bottled and sachet water on this ladder presented a unique problem, which was resolved via the backup water source. Our survey of DHS country reports revealed that until about 2008, bottled and sachet water were classified as either improved or unimproved, depending on how the backup water source was classified. However, starting in 2010, the backup source question was dropped from the DHS questionnaire. (The backup source was asked only in the case of Ethiopia (ET), but the DHS report provides no evidence to suggest that this information was ultimately used to classify bottled/sachet water one way or another.) Across all countries, bottled water was classified as an improved source. Sachet water was not listed as an option, except in two countries - GH, where it was classified as an improved source and NG, where it was classified as an unimproved source. A summary of the DHS categorization of water sources is provided in Table S1 (available with the online version of this paper).

Despite the change in DHS classification of bottled and sachet water, JMP has followed the 'backup-dependent' approach where bottled and sachet water are classified as improved or unimproved, depending on the nature of the backup source. However, the JMP's approach toward packaged water is likely to change as the global water monitoring shifts from the Millennium Development Goals (MDG) paradigm to the Sustainable Development Goals (SDG). Given the status of DHS as a premier international survey and its role in setting priorities for funding, interventions, and policy changes by its parent body and the world's leading government donor, USAID, modifications to the assessment of water sources by DHS is an important topic worthy of discussion.

Classification of bottled water as an improved source, regardless of the backup source, along with ad hoc changes to the classification of sachet water introduces variability to the process of tracking water quality metrics. To highlight and quantify the variability in tracking water quality metrics introduced by technical changes to the classification of bottled and sachet water, we create four scenarios where packaged water components (bottled, sachet, and refill) are variously classified as improved or unimproved, and then observe changes to the population relying on improved water sources. Furthermore, we focus on one of the study geographies to identify factors associated with packaged water use.

## METHODS

We relied on data collected in nine study geographies across eight countries - Burkina Faso (BF), DR Congo (Kinshasa; CDK), ET, GH, Indonesia (ID), Niger (Niamey, NEN), NG (Kaduna state; NGK), NG (Lagos state; NGL), and Uganda (UG) - by the PMA2020 (http://pma2020.org), a large-scale monitoring program led by Johns Hopkins University (JHU). PMA2020 surveys are modular in design, with a core set of questions repeated over time and across countries for easy comparison. Currently, PMA2020 surveys have a family planning and a water, sanitation and hygiene (WASH) module. Data and results presented in this article are derived from the drinking water section of the WASH module. The surveys were approved by the institutional review boards in each partner institution (listed in the Acknowledgments) and at JHU.

In addition to asking respondents to identify the primary water source (main option) for drinking purposes, PMA2020 interviews require respondents to indicate all sources used by the household on a regular basis for any part of the year for any purpose (regular option). Data on regular options provide insight into a household's decision-making on source-switch-ing when the main source is highly unreliable or unavailable for part of the year. Vedachalam *et al*. (2017) used PMA2020 data to reveal underreporting of high-risk water and sanitation practices across several countries in Africa and Asia.

For the purposes of this article, data from multiple rounds were aggregated, where possible, to generate a single dataset for each country. Only complete and *de jure* respondents (usual household members based on the roster provided by the partner country's government agency and does not include individuals who are typically not part of the HH, but present at thetimeofinterview)wereincludedforanalysis,whichwas conducted using Stata v14.1 (StataCorp 2015). For each study geography, estimates for the main and regular use of packaged water (bottled and refill in ID; bottled and sachet in the rest) along with the 95% confidence interval (CI) were calculated. Study areas where packaged water constituted one of the regular options for at least 5% of the respondents were selected for further examination. The evaluation of packaged water use in these study geographies was based on data from 112,083 respondents. The breakdown by region was as follows: 21,596 (CDK); 30,483 (GH); 45,006 (ID); 11,401 (NGK); and 3,597 (NGL).

In each of the selected geographies, the proportion of residents relying on unimproved water sources was calcu-lated under four scenarios:

1. *Ideal* scenario: Both bottled water and sachet/refill water are improved. This scenario assumes that the packaged water industry is well-regulated at the country and regional level, ensuring a high quality of source water and effective monitoring of the packaging and distribution process.
2. *Backup-dependent* scenario: Determination of improved/ unimproved is based on the backup source. This scenario is the pre-2008 DHS paradigm, and presumes that it is difficult to assess the quality of the source, and hence relies on the backup source to classify packaged water as improved or unimproved.
3. *Hybrid* scenario: Bottled water is improved; sachet/refill water is unimproved. This is a hybrid scenario, where only bottled water is assumed to be an improved source. This might be plausible because (i) bottled water is regulated under stricter laws in several countries, (ii) multinational companies in the bottled industry may follow uniform sourcing and production checks, and (iii) higher pricing of bottled water compared to sachet/refill water allows the manufacturers to spend more on quality control.
4. *Worst-case* scenario: Both bottled water and sachet/refill water are unimproved. This is the opposite of the *ideal* scenario, and assumes a poorly regulated packaged water industry, where the source water quality is not guaranteed, and the packaging and distribution is not monitored.

DHS classification of packaged water in GH follows the *ideal* scenario, while that in NG follows the *hybrid* scenario. The backup-dependent scenario was employed by DHS in every country until 2008. The *worst-case* scenario is the only truly hypothetical scenario, but one which might be applicable in countries with poor bottling regulations and monitoring.

Following development of the scenarios, we utilized household data from one of the selected study geographies to build binary logistic (logit) regression models to study the socio-economic and structural factors associated with packaged water use, both as the main option and as one of the regular options. The dependent variable in each of the models was the use of packaged water. Independent explanatory variables included socio-economic characteristics, structural factors, and a geographic regional control. Socio-economic variables included a dummy for urban location of the household, normalized wealth score, and household size. Structural factors included number of water sources, reliability of the main source for the non-packaged water users, or the reliability of the main alternative for packaged water users. Estimates are presented as odds ratios, with cluster-robust standard errors. Clustering is performed at the level of enumeration areas (EAs), which are clusters of about 200 households.

## RESULTS

**Figure 2:**
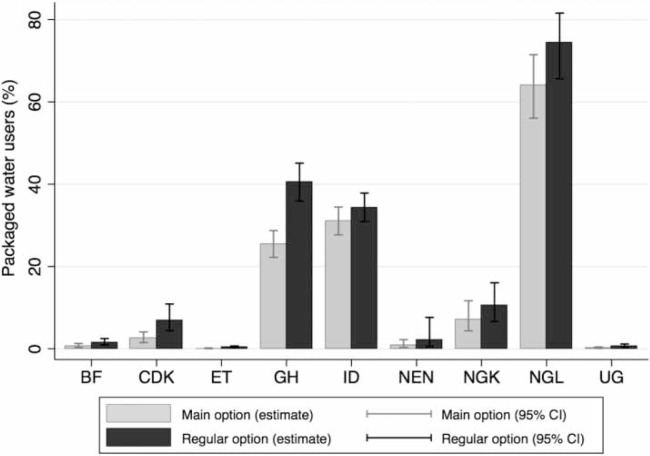
Use of packaged water for drinking needs in nine study geographies: Burkina Faso (BF), DR Congo (Kinshasa; CDK), Ethiopia (ET), Ghana (GH), Indonesia (ID), Niger (Niamey, NEN), Nigeria (Kaduna state; NGK), NG (Lagos state; NGL), and Uganda (UG).

In the nine PMA2020 study geographies, use of packaged water varied widely. Packaged water use as the main option ranged from 0.1% in ET to 65% in NGL (Figure 2). When considering all regular options, packaged water use ranged from 0.4% in ET to 75% in NGL. Across all study geographies, packaged water use as a regular option exceeded its use as the main option - by as little as 0.4% in ET and as much as 15% in GH.

Based on these results, we selected geographies where packaged water constituted one of the regular options for at least 5% of the users. The selected geographies included CDK, GH, NGK, NGL, and ID. As observed from Figure 2, sachet water is especially popular in GH and NG. However, in NG, the states of Kaduna and Lagos exhibited markedly different profiles in their consumption of packaged water. More than half of Lagos state residents consumed packaged water as their main option, of which, 7% were bottled water users. Kaduna residents, on the other hand, consumed packaged water at a much lower rate, and bottled water was used by less than 1% of the residents. ID was also unique in its notable consumption of refill water. Sachet water was not presented as an option to ID survey respondents.

**Figure 3:**
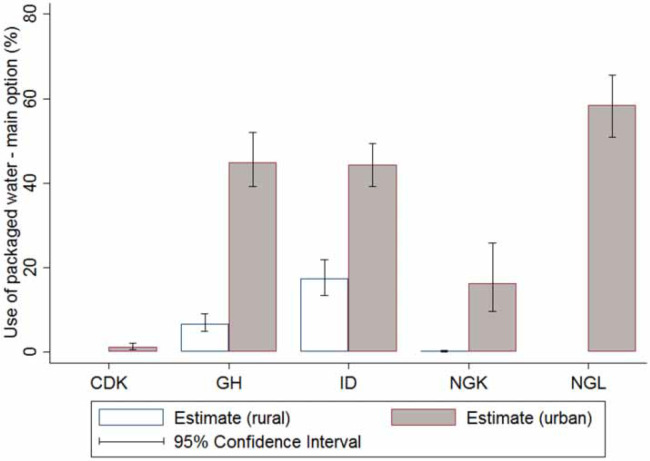
Packaged water use as the main option stratified by location, in DR Congo (Kinshasa; CDK), Ghana (GH), Indonesia (ID), Nigeria (Kaduna state; NGK), and Nigeria (Lagos state; NGL). CDK and NGL sampling frame was restricted to urban respondents.

**Figure 4:**
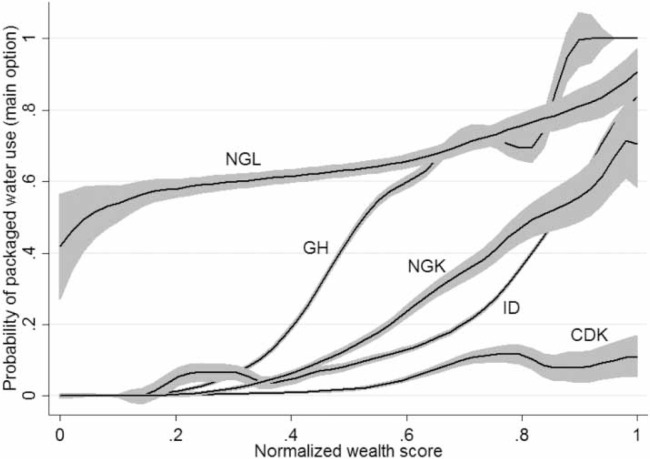
Local polynomial smoothing functions depicting packaged water use (main option) over normalized wealth score, in DR Congo (Kinshasa; CDK), Ghana (GH), Indonesia (ID), Nigeria (Kaduna state; NGK), and Nigeria (Lagos state; NGL). *Note*: Raw wealth scores for each study geography are normalized to range between 0 and 1 to ensure ease of overlay and comparison.

We next explored the relationships between the use of packaged water and socio-economic characteristics such as location and wealth. In the three study geographies that had both urban and rural samples, packaged water use as the main option was consistently higher in the urban sample (Figure 3). A locally weighted polynomial regression of packaged water use as the main option against normalized wealth score shows consumption increasing over wealth (Figure 4). In all geographies except NGL, packaged water consumption was extremely low in poor households and increased gradually with rising wealth. Packaged water consumption started high in poor NGL households and rose only marginally across the wealth spectrum, revealing the important role played by packaged water in all NGL households, regardless of wealth. The plot of packaged water use as a regular option against normalized wealth score differed in magnitude but was characteristically similar to the one observed in Figure 4 (see Supplementary information, Figure S1, available with the online version of this paper).

**Figure 5:**
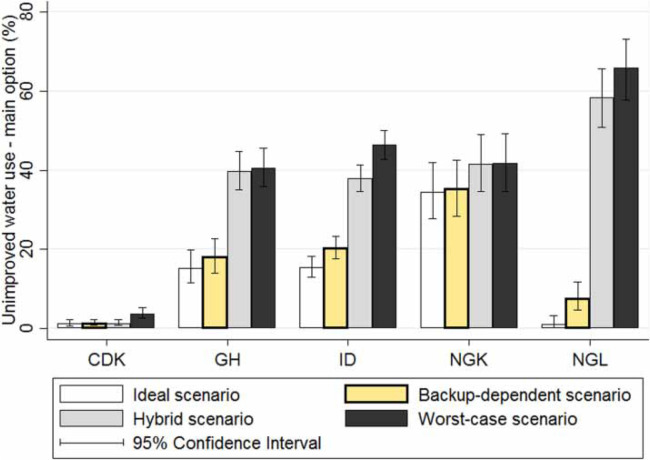
Use of unimproved water as the main drinking option under four scenarios, in DR Congo (Kinshasa; CDK), Ghana (GH), Indonesia (ID), Nigeria (Kaduna state; NGK), and Nigeria (Lagos state; NGL). As per the current DHS methodology, GH follows the ideal scenario, NGK and NGL follow the hybrid scenario, while sachet water is not classified explicitly as improved or unimproved in other regions (CDK and ID).

Presented in Figure 5 is the percent of respondents in the five selected study geographies that consume unimproved water as their main option under each of the four scenarios described in the Methods section. Overall, estimates for unimproved water consumption were markedly higher in the *hybrid* and *worst-case* scenarios, as compared to *ideal* and *backup-dependent* scenarios. The magnitude of difference between the two groups of scenarios was based on the fraction of respondents reliant on sachet/refill water as their main option. Estimates for unimproved water consumption as a regular option were similar in pattern, although not in magnitude, to those seen in Figure 5 (see Supplementary information, Figure S2, available with the online version of this paper).

We next focused on one of the study geographies, GH, to investigate the role of socio-economic factors in the use of packaged water. We already observed that packaged water use was higher in urban and wealthier households (Figures 3 and 4). However, urban households in LMICs are typically also wealthier, so a regression model was used to isolate the effect of each explanatory variable. Variables and their summary statistics are described in Table 1. Panels A and B in Table 2 show logistic regression models where the dependent variables are packaged water use as the main and regular options, respectively.

**Table 1:** Variable definitions and summary statistics

Variable	Description	Min	Max	Mean	Std. dev	Median
Packaged_main	Packaged water use as the main option	0	1	0.25	0.43	0
Packaged_regular	Packaged water use as the regular option	0	1	0.40	0.49	0
Urban	Location of the household; 1 if urban; 0 otherwise	0	1	0.48	0.50	0
Wealth score	Index of household wealth derived from ownership of select assets	−6.24	10.18	0.04	2.67	−0.01
Household size	Number of household members	1	36	6.56	4.70	5
Water sources	Number of water sources	1	7	2.13	1.06	2
Water reliability	Reliability of the main option; reliability of the primary alternative if packaged water is the main option					
1. Always		0	1	0.56	0.50	1
2. Predictably intermittent		0	1	0.22	0.41	0
3. Unpredictable		0	1	0.22	0.41	0

**Table 2:** Logistic regression results for use of packaged water as the main and regular options

Variable	Panel A: Main	Panel B: Regular
Urban	3.56^***^ [2.09, 6.07]	1.01 [0.44, 2.32]
Wealth score	1.51^***^ [1.39, 1.65]	1.61^***^ [1.28, 2.01]
Household size	0.90^***^ [0.85, 0.96]	0.89^***^ [0.85, 0.94]
Water sources	2.93^***^ [2.29, 3.75]	18.77^***^ [10.59, 33.26]
Water reliability		
1. Always	–	–
2. Intermittent	0.62^**^ [0.39, 0.99]	0.72 [0.38, 1.38]
3. Unpredictable	0.90 [0.62, 1.32]	0.80 [0.36, 1.81]
Constant	0.01^***^ [0.003, 0.02]	0.001^***^ [0.0004, 0.005]
Region control	Yes	Yes
Clustering effect	Yes	Yes
N	28,883	22,845
Pseudo R^2^	0.48	0.61

Notes: Parameter estimates are odds ratios, with 95% CI in brackets. Region control refers to the ten sub-national regions officially recognized by the Government of GH. Panel B excludes observations with only one water source, hence N is lower than that in Panel A. Standard errors are clustered by EA.

* p < 0.10

** p < 0.05

*** p < 0.01.

Packaged water use as the main option (panel A) was more likely to be observed in urban (OR = 3.56; 95% CI [2.09-6.07]) and wealthier households (1.51; 1.39-1.65) even after controlling for several other socio-economic variables. Smaller households (0.90; 0.85-0.96) and access to multiple sources (2.93; 2.29-3.75) were also associated with packaged water use. Reliability of the water source was associated with packaged water use in an unexpected manner. In comparison with an always-reliable main option, users with access to an intermittently predictable (0.62; 0.55-0.70) source were less likely to use packaged water, while access to an unpredictable source as the main option was no different than having an always-reliable source. When all regular sources were included, drivers of packaged water use behavior (panel B) remained largely similar to those observed in panel A. Wealthy (1.61; 1.28-2.01), and smaller (0.89; 0.85-0.94) households and those with access to multiple water sources (18.77; 10.59-33.26) were most likely to regularly rely on packaged water. Household location and source reliability, however, were no longer drivers of packaged water use.

## DISCUSSION

The popularity and widespread usage of packaged water in many LMICs may be attributed to convenience, safety, and attractive marketing of the product itself, or to the poor public infrastructure. In the PMA2020 survey of nine study geographies, packaged water consumption varied but constituted a regular source for more than 5% of the residents in five of those geographies. NGL's urban sampling frame could be a reason for the large packaged water use when compared to the nationally representative samples of GH and ID. However, NGL points to the outsize role packaged water often plays in an urban water landscape, where a highly mobile population can afford to pay for easy access to water, yet chronically suffers from lack of a reliable water service.

PMA2020's inclusion of multiple water sources in its survey is a departure from other accepted global monitoring programs such as DHS and JMP that only assess the main source. As a result, estimating the regular use of packaged water is a better indicator of overall packaged water consumption than merely estimating its use as the main option. In every study geography, regular users of packaged water outnumbered the main users, in some cases by a significant amount.

In study geographies that had both rural and urban sampling frames (GH, ID, and NGK), urban consumption of packaged water was far higher than that in rural locations, suggesting packaged water use as, while not exclusively, a predominantly urban phenomenon. We also observed packaged water use was influenced by wealth. In all five study geographies, the proportion of packaged water users increased along with rising wealth, although there were differences in the profile of users along the wealth spectrum.

The hypothetical classification of packaged water (or its components) as either improved or unimproved sources provided meaningful insights into the role of packaged water in the drinking water landscape in the selected geographies. The use of backup source to determine the nature of packaged water source (*backup-dependent* scenario) is an alternative way to factor in the uncertainty associated with the quality of the packaged water source. Literature showing poor quality sachet water sold in NG and GH provides a plausible reason for such a cautious approach. After following the *backup-dependent* scenario for many years, DHS switched to a scenario that can, at best, be labeled ad hoc. While one may assume DHS was prompted to undertake this change based on evidence from the countries in question, there is no formal record of a country-level decision (Fred Arnold, ICFI, personal communication, December 4, 2015).

Estimates for unimproved water if packaged water is classified as an improved source (*ideal* scenario) were closer to, but always lower than that under the *backup-dependent* scenario. As mentioned earlier in the Introduction section, even as DHS treats packaged water variously in different countries, JMP has consistently used the *backup-dependent* scenario for MDG reporting. However, JMP's treatment of packaged water will change to the ideal scenario under the SDG reporting (UNICEF/WHO 2016). The difference between these two scenarios is marginal in some cases (CDK and NGK), but moderate (GH and ID), or very significant (NGL) in others.

DHS treats packaged water in GH under the *ideal* scenario, although it is not clear if there is evidence to support this distinction. Distribution and point-of-use sale of sachet water in GH follows a similar trajectory to that observed in NG (Boakye-Yiadom 2013). Despite existing regulations and enforcement from regulators such as the GH Standards Authority and NG's National Agency for Food and Drug Administration (NAFDAC), many suppliers often fail to meet standards, putting public health at risk (Akunyili 2003; Premium Times 2013). It may be more reasonable then to assume the *worst-case* scenario where packaged water is treated as an unimproved source. Under this scenario, the proportion of residents relying on unimproved water sources increases significantly. Even a weaker assumption, that bottled water is an improved source while sachet/refill water is not (*hybrid* scenario), yields results that are similar to that under the *worst-case* scenario. This may be explained by the fact that bottled water is a much smaller component of the packaged water mix in all study geographies except CDK.

Regression analysis of packaged water users in GH revealed that they are more urban and wealthier than users of other drinking water sources, even after controlling for other variables. This suggests that even as packaged water use has become popular in GH, it is mostly used by the wealthy and urban section of the population, leaving the rest of the country with poor, often unsafe alternatives. Smaller household size and access to multiple water sources also resulted in a higher likelihood of packaged water use. Location and source reliability were the only variables where regular users of packaged water differed from the main users. The counter-intuitive role played by reliability of the main water source in the 'main option' model and the lack of significance in the 'regular option' model suggests that users do not always rely on packaged water as an alternative to poor choices but rather to supplement existing reliable water sources. This finding may warrant further investigation as reliability may be closely correlated with other underlying factors that are not captured in our models.

## CONCLUSIONS

Packaged water use has increased in many LMICs where piped water availability is at best, unreliable and at worst, non-existent. Packaged water, but more so, sachet water, has filled the unmet need for an easy and accessible water source. The PMA2020 surveys identified high packaged water use in five study areas with NGL recording the highest use. Analysis of packaged water use in study geographies with both urban and rural samples portrays packaged water as primarily an urban and wealthy consumer product. Based on an analysis of Ghanaian respondents, this relationship held even after controlling for other socio-economic factors.

Even though safety of any water source, including piped supply, is hard to guarantee without strict controls and a monitoring program, packaged water is especially ripe for contamination during production and distribution. A more comprehensive look at the role of sachet water in the larger water supply landscape suggests possible public health benefits. The mixed evidence points to the quandary of whether to place sachet water on the water ladder as an improved source or not. The development of four hypothetical scenarios played on this ambiguity, and demonstrated how classifying a certain source as improved or not plays a critical role in the overall metric that indicates progress at the national and international level.

The decision of DHS to classify sachet water as improved in GH and unimproved in NG signifies the need for country-level decisions on progress indicators that better capture the quality of water infrastructure provided. At the same time, the DHS decision also suggests that moving sources from one category to another ad hoc can lead to a certain desired outcome. DHS and JMP metrics drive national and institutional donor policies; consequently, care must be taken to ensure that the metrics and classification schema reflect the needs of the people they are designed to help.
