# Supplementary material for: The role of packaged water in meeting global targets on improved water access
Source: J Water Sanit Hyg Dev. 2017 Apr 7;7(3):369–77. doi: 10.2166/washdev.2017.155 (PMC7734372; doi:10.2166/washdev.2017.155)
Supplement: Supplementary file 1 [file JWSHD-07-03-369-s001.pdf]

Supplementary information

Table S1 | DHS classification of drinking water sources as improved or unimproved

**Drinking water source**	**BF (2010)**	**CD (2013)**	**ET (2011)**	**GH (2014)**	**ID (2012)**	**NE (2012)**	**NG (2013)**	**UG (2011)**
Piped into dwelling								
Piped to yard								
Public tap/standpipe								
Borehole								
Protected spring								
Rainwater								
Bottled water								
Sachet water								
Unprotected well								
Unprotected spring								
Tanker								

truck/cart								
Surface water								

Notes: Columns are country names abbreviated as follows: Burkina Faso (BF), DR Congo (CD), Ethiopia (ET), Ghana (GH), Indonesia (ID), Niger (NE), Nigeria (NG), and Uganda (UG). Improved sources of water are shaded in light gray, while unimproved sources are shown in dark gray. While the DHS classification of sources is consistent across most countries included in our study, protected spring and rainwater are classified as unimproved sources in Indonesia. Also, sachet water is not presented as an option to survey respondents in most countries (shaded white). The only exceptions are GH and NG, where it is classified as an improved and an unimproved source, respectively.

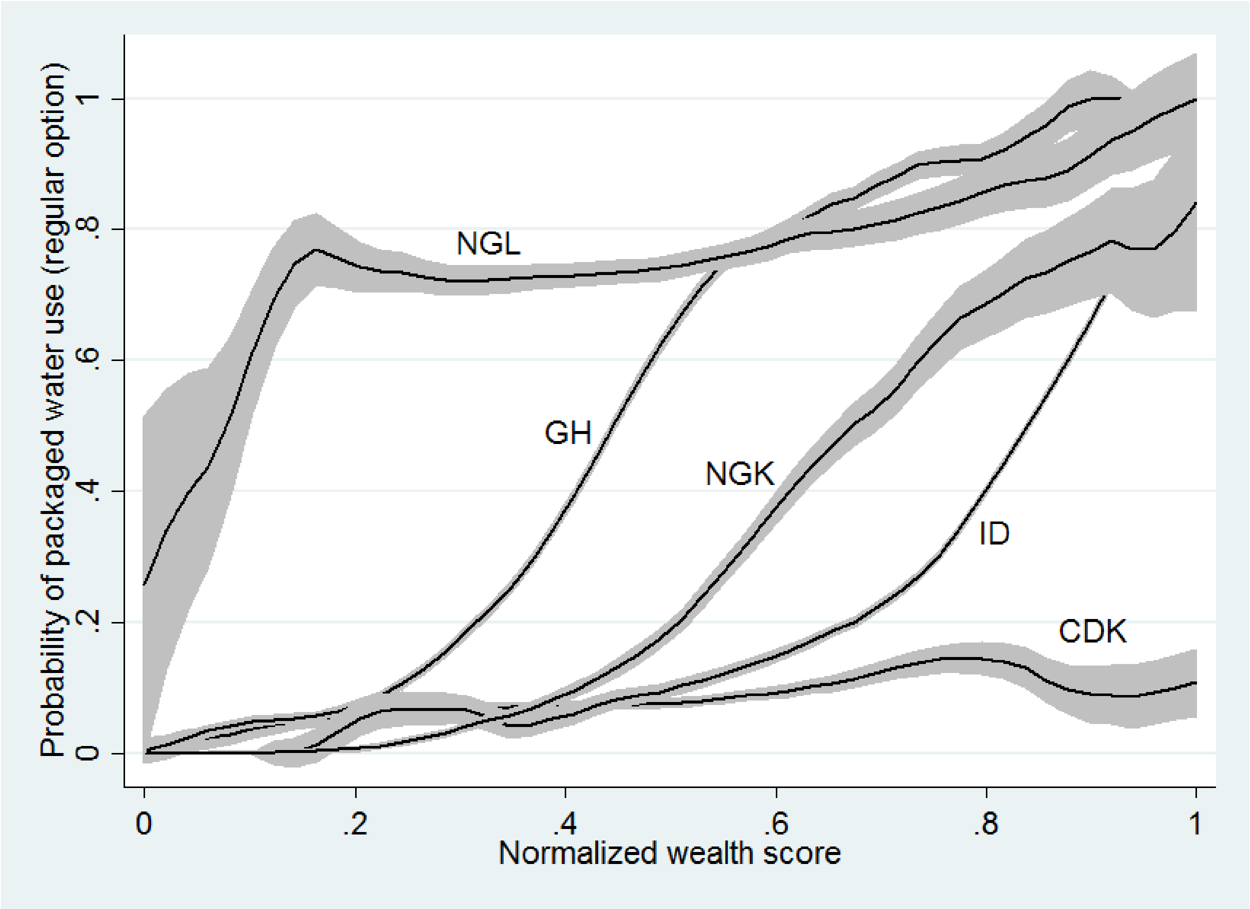

Figure S1 | Local polynomial smoothing functions depicting packaged water use (regular option) over normalized wealth score in DR Congo (Kinshasa; CDK), Ghana (GH), Indonesia (ID), Nigeria (Kaduna state; NGK), and Nigeria (Lagos state; NGL). Note: Raw wealth scores for each study geography are normalized to range between 0 and 1 to ensure ease of overlay and comparison.

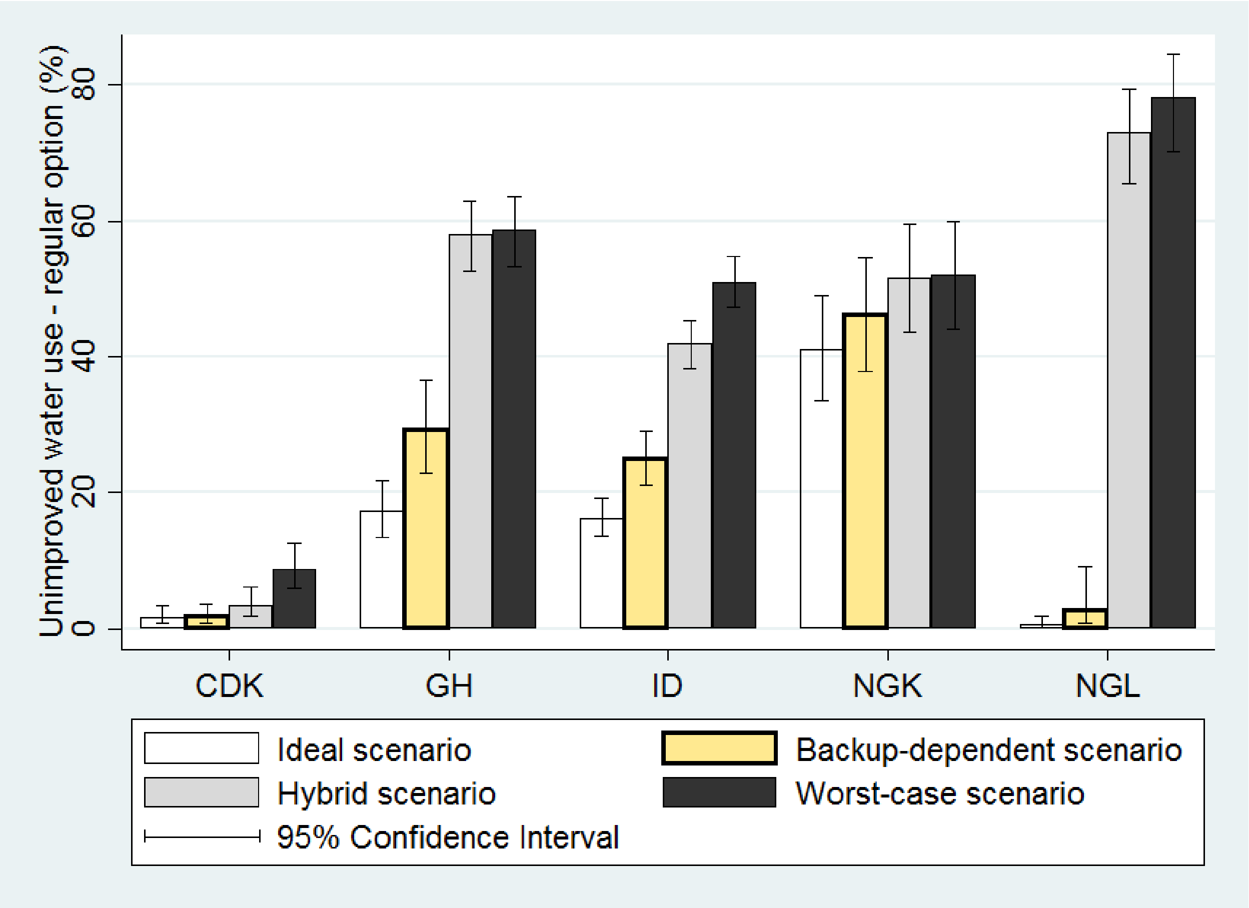

Figure S2 | Use of unimproved water as a regular drinking option under four scenarios, in DR Congo (Kinshasa; CDK), Ghana (GH), Indonesia (ID), Nigeria (Kaduna state; NGK), and Nigeria (Lagos state; NGL). As per the current DHS methodology, GH follows the ideal scenario, NGK and NGL follow the hybrid scenario, while sachet water is not classified explicitly as improved or unimproved in other regions (CDK and ID).
